# Reactive Oxygen Species-Induced Lipid Peroxidation in Apoptosis, Autophagy, and Ferroptosis

**DOI:** 10.1155/2019/5080843

**Published:** 2019-10-13

**Authors:** Lian-Jiu Su, Jia-Hao Zhang, Hernando Gomez, Raghavan Murugan, Xing Hong, Dongxue Xu, Fan Jiang, Zhi-Yong Peng

**Affiliations:** ^1^Department of Critical Care Medicine, Zhongnan Hospital of Wuhan University, Wuhan, 430071 Hubei Province, China; ^2^Center of Critical Care Nephrology, Department of Critical Care Medicine, University of Pittsburgh Medical Center, Pittsburgh, 15223 PA, USA

## Abstract

Reactive oxygen species- (ROS-) induced lipid peroxidation plays a critical role in cell death including apoptosis, autophagy, and ferroptosis. This fundamental and conserved mechanism is based on an excess of ROS which attacks biomembranes, propagates lipid peroxidation chain reactions, and subsequently induces different types of cell death. A highly evolved sophisticated antioxidant system exists that acts to protect the cells from oxidative damage. In this review, we discussed how ROS propagate lipid peroxidation chain reactions and how the products of lipid peroxidation initiate apoptosis and autophagy in current models. We also discussed the mechanism of lipid peroxidation during ferroptosis, and we summarized lipid peroxidation in pathological conditions of critical illness. We aim to bring a more global and integrative sight to know how different ROS-induced lipid peroxidation occurs among apoptosis, autophagy, and ferroptosis.

## 1. Introduction

Reactive oxygen species (ROS) are produced by normal physiological processes and play important roles in cell signaling and tissue homeostasis [1]. However, excess radical species produce adverse modifications to cell components and augment various pathogenesis, such as lipids, proteins, and DNA damage [2]. Cellular membranes or organelle membrane, due to their high polyunsaturated fatty acids (PUFAs), are especially susceptible to ROS damage, which is called “lipid peroxidation.” Lipid peroxidation is a process in which free radical species such as oxyl radicals, peroxyl radicals, and hydroxyl radicals remove electrons from lipids and subsequently produce reactive intermediates that can undergo further reactions. The lipid peroxidation damages phospholipids directly and can also act as cell death signal which induces programmed cell death. Oxidized phospholipids can also play an important role in many inflammatory disease and frequently mediate proinflammatory change [3]. Recently, ferroptosis, a new form of programmed cell death, has been found to be caused by lipid peroxidation [4], which highlights lipid peroxidation during the physiological process of cell death. It is therefore of great interest to understand how ROS is produced and eliminated and how ROS-induced lipid peroxidation contributes to cell death. In this review, we summarize the processes of ROS-induced lipid peroxidation among apoptosis, autophagy, and ferroptosis and discuss how they come together to affect the fate of a cell in a more global and integrative way.

## 2. Generation of ROS and Antioxidant System

### 2.1. Generation of ROS

**Figure 1:**
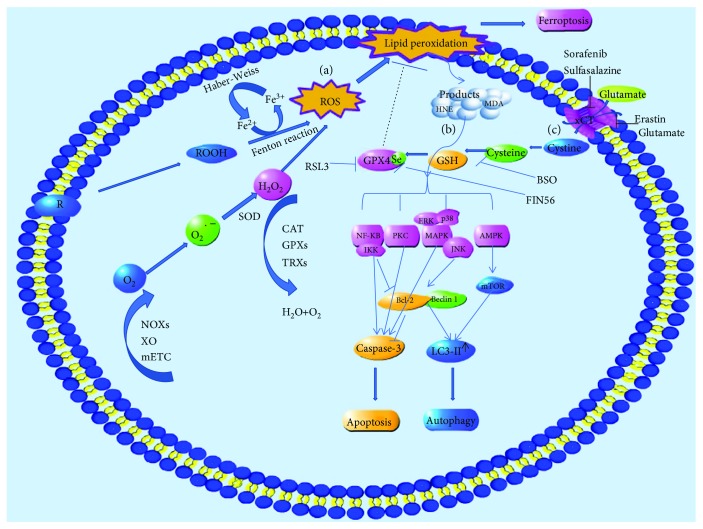
Generation of ROS and lipid peroxidation in cell death. (a) Generation of ROS; ROS are derived from superoxide radical, whose formation is mainly through NADPH oxidases, xanthine oxidase, and the mitochondrial electron-transport chain. Polyunsaturated fatty acids containing phospholipids can generate alkoxyl (RO^·^) radicals by Fenton chemistry reaction. (b) The products of lipid peroxidation induce apoptosis and autophagy via different pathways. (c) GPX4 activity decreases and a depletion of GSH causes lipid peroxidation and consequently to ferroptosis.

ROS are partially reduced oxygen-containing molecules, which are free radicals and/or oxygen derivatives, including superoxide anion, hydrogen peroxide, hydroxyl radical, lipid hydroperoxides, and peroxyl radicals. Most intracellular ROS are derived from superoxide radical, whose formation is mainly through NADPH oxidases (NOXs), xanthine oxidase (XO), and the mitochondrial electron-transport chain (mETC) in endogenous biologic systems [5, 6]. ROS are converted to hydrogen peroxide by the superoxide dismutase (SOD) and yield the highly toxic hydroxyl radical in the presence of reduced iron (Fe^2+^) through the Fenton reaction which have different peroxide species to generate hydroxyl (^·^OH) or alkoxyl (RO^·^) radicals [7]. Ferric iron (Fe^3+^) can be recycled to Fe^2+^ via the *Haber-Weiss* reaction by oxidation with a peroxyl radical to oxygen [8, 9] (Figure 1). Imbalance in the rate of ROS generation leads to oxidative stress and consequent production of free radicals that can damage DNA, proteins, and lipids [10].

### 2.2. Antioxidant System

**Figure 2:**
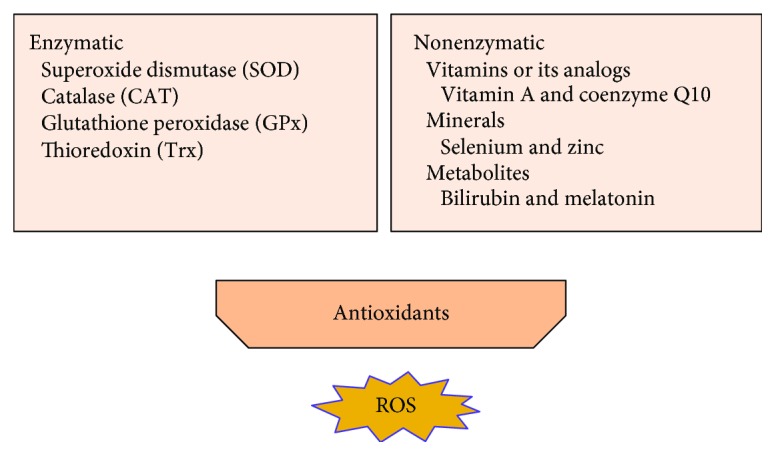
Antioxidant system protects cell from ROS-induced lipid peroxidation.

Antioxidants can counteract free radicals and neutralize oxidants. The general endogenous antioxidant system consist of (1) enzymatic antioxidants like superoxide dismutase (SOD), catalase (CAT) and glutathione peroxidase (GPx), and thioredoxin (Trx); and (2) nonenzymatic antioxidants that include vitamins or its analogs (vitamins A, C, and E; coenzyme Q10; and flavonoids), minerals (selenium and zinc), and metabolites (bilirubin and melatonin) (Figure 2).

#### 2.2.1. Enzymatic Antioxidants

The enzymatic antioxidants have an effective protective effect against oxidative attack due to the ability to decompose ROS [11]. Among them, SOD is very important for living cell because most of ROS is produced from superoxide. SOD can catalyze the conversion of superoxide into oxygen and hydrogen peroxide [12]. CAT can decompose the hydrogen peroxide into molecular oxygen and water. CAT significantly reduced oxidative stress and restored mitochondrial structure by enhancing the mitochondrial membrane potential (*Δψ*m) so as to play an antiapoptotic effect and normalize replicative and wound healing capacity [13, 14]. Glutathione peroxidases (GPxs) consist of multiple isoenzymes with distinct subcellular locations exhibiting different tissue-specific expression patterns [15, 16]. During persistent oxidative stress, GPxs become the main H_2_O_2_-scavenging enzymes after ascorbate peroxidases being inhibited [17]. GPxs detoxify other toxic organic hydroperoxides by catalyzing the reduction of H_2_O_2_ and hydroperoxides to water or alcohols [16]. The thioredoxin (Trx) antioxidant system is composed of NADPH, thioredoxin reductase (TrxR), and Trx. Trx and TrxR catalyze the NADPH-dependent reduction of the active-site disulfide in oxidized Trx to give a dithiol in reduced Trx. NADPH maintains CAT in the active form and is used as a cofactor by TRX and GSH reductase, which converts glutathione disulfide (GSSG) to glutathione (GSH), a cosubstrate for the GSH-Pxs. The reaction sequence of TrxR-mediated reduction of protein disulfides has been reviewed [18]. The thioredoxin (Trx) and glutathione (GSH) systems could be complementary to each other. The TrxR1/Trx1 system can sustain reduced GSH pools in the absence of glutathione reductase [19].

#### 2.2.2. Nonenzymatic Antioxidants

Vitamin A (retinol) is a carotenoid synthesized in the liver and resulted from the breakdown of *β*-carotene. Vitamin A can directly interact with peroxyl radicals forming free carbon-centered radical adducts and scavenge peroxyl radicals by electron transfer before they propagate peroxidation to lipids [20]. Coenzyme Q10 (CoQ10) is the single lipophilic antioxidant which is essential for electron transport during mitochondrial respiration. It was reported to prevent oxidative damage of lipid peroxyl radicals and improve mitochondrial biogenesis [21, 22]. Vitamin C (ascorbic acid) can scavenge a variety of oxygen free radicals [23]. Vitamin E (a-tocopherol) is a fat-soluble antioxidant that can protect the polyunsaturated fatty acids (PUFAs) in the membrane from oxidation, regulate the production of ROS, and modulate signal transduction [24]. Flavonoids are extensively distributed in beverages, vegetables, and fruits which have antioxidant activity by inhibiting the enzymes responsible for superoxide production as well as NADH oxidase [25]. Selenium and zinc have the antioxidative function due to their activity maintenance of many enzymes. Zinc acts in the stabilization of membranes by inhibiting NADPH-oxidase and inducing the synthesis of metallothioneins, and it is also a component of SOD [26]. Selenium serves as a structural and catalytic cofactor for numerous proteins such as GPxs and thioredoxin reductase (TrxR) which is an important component of enzymatic antioxidants. Many metabolites such as bilirubin and melatonin have an antioxidative function. Evidence suggests that bilirubin possesses antioxidant properties. Bilirubin treatment can inhibit the TLR4-mediated upregulation of iNOS by preventing activation of hypoxia inducible factor-1*α* (HIF-1*α*) through scavenging of NOX-derived ROS [27]. Melatonin improves the intramitochondrial antioxidative defense by enhancing reduced glutathione levels and inducing glutathione peroxidase and superoxide dismutase to inhibit peroxidation [28]. Melatonin may prevent mitochondrial damage for it behaves like synthetic mitochondrion-targeted antioxidants which concentrate in mitochondria at relatively high levels [29].

## 3. Production of Lipid Peroxidation and Its Detecting Methodologies

ROS generation in the biomembranes is very high due to the solubility of molecular oxygen. Thus, the membrane phospholipids, containing high levels of PUFAs, are extremely sensitive to be attacked by ROS [30]. Moreover, the PUFAs themselves convert into reactive free radicals after reacting with the free radicals which are able to propagate lipid peroxidation chain reactions [31].

### 3.1. Production of Lipid Peroxidation

The products of lipid peroxidation chain reactions display high biological activity [32]. It destroys DNA, proteins, and enzyme activity as well as acts as molecular to activate signaling pathways initiating cell death [33]. Biomembranes are prone to undergo lipid peroxidation, and it is possibly via two pathways: nonenzymatic and enzymatic.

#### 3.1.1. Nonenzymatic Autoxidation

**Figure 3:**
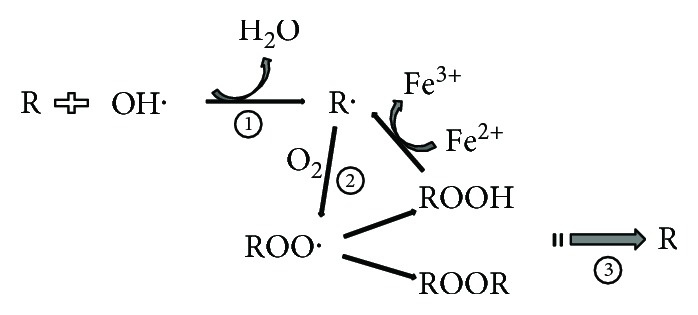
Nonenzymatic autoxidation of polyunsaturated fatty acids. R is polyunsaturated fatty acids containing phospholipids; R^·^ is an alkoxyl radical; ROO^·^ is a peroxyl radical (ROO^·^); ROOH is a lipid hydroperoxide (ROOH); ROOR is PL-OO^·^ to the bis-allylic position of another PL to form PL-OO-PL^·^ dimers; ① means initiation stage; ② means propagation stage; ③ means termination stage.

The nonenzymatic pathway which is also called “nonenzymatic phospholipid (PL) autoxidation” is iron-dependent lipid peroxidation. Autoxidation radical chain reactions of PUFA containing PLs can be divided into three stages: initiating—polyunsaturated acyl chain of a PL is oxidized to generate R^·^ (a carbon-centered radical containing PL) by losing hydrogen to hydroxyl (^·^OH); propagating—R^·^ readily reacts with molecular oxygen to form a peroxyl radical (R-OO^·^) [34]. On the one hand, propagation reactions of R-OO^·^ include hydrogen abstracting from a PL molecule to form a lipid hydroperoxide (R-OOH). On the other hand, addition of R-OO^·^ to the bis-allylic position of another PL forms R-OO-R^·^ dimers [35]. During the process of Fenton chemistry, R-OOH can undergo reductive cleavage to generate alkoxyl (RO^·^) radicals [36]. Autoxidation reactions of PUFAs form many electrophilic species such as malondialdehyde, isoprostanes, and 4-hydroxy-2-nonenal (4-hydroxy-2,3-trans-nonenal, HNE). These products of lipid peroxidation have various biological functions [37]. The last stage is terminating—two radicals of the chain reaction react with each other to form stable molecules and the antioxidants effectively decompose radicals which inhibit the chain reaction [38] (Figure 3).

#### 3.1.2. Enzymatic PL Peroxidation

Enzymatic peroxidation is catalyzed by lipoxygenase (LOX) which can also participate in the formation of R-OOH. Lipid peroxidation involves a highly organized oxygenation center, wherein oxidation occurs on only one class of phospholipids [39]. Arachidonic (C20: 4) and linoleic (C18: 2) are the most abundant polyenoic fatty acids that serve as substrates for LOX using molecular oxygen to form hydroperoxyl groups at different carbon position of acyl chains [40]. Arachidonate lipoxygenase-15 (Alox15) which encodes for the 12/15-LOX has a unique substrate requirement among the LOX family. It can directly oxygenate PUFA containing PLs without prior release of esterified PUFA by phospholipase A2 (PLA2) [41]. Alox5 and Alox12 which encodes for a 5-lipoxygenating and enzyme platelet-type 12-LOX, respectively, have been shown to provide oxygenated acyl precursors to generate oxygenated PLs [42, 43].

### 3.2. Detecting Methodologies

Various methods have been applied to the measurement of oxygen radicals and their damaging effects on membrane lipids. Hydroperoxides, primary product of lipid peroxidation, are not stable which may include phospholipid hydroperoxides [44]. An HPLC-chemiluminescence (HPLC-CL) detection method has been developed by which these species can be measured [45]. The main aldehyde product of lipid peroxidation is the 3-carbon dialdehyde species malondialdehyde (MDA) and 4-hydroxy-2-nonenal (HNE) [46]. MDA can be measured by the thiobarbituric acid (TBA) test using UV/visible spectrophotometry. Immunoblotting or immunohistochemistry is available for measuring 4-HNE/protein adducts [47]. Isoprostanes (IsoP) are a series of prostaglandin-like compounds produced by a free radical-mediated lipid peroxidation of arachidonic acid independent of cyclooxygenase. Utilizing gas chromatography/mass spectrometry (GC/MS) with negative ion chemical ionization, the lipid peroxidation-derived 5- and 15-F2t isoprostanes (8-isoprostaglandin F 2*α*) can be accurately measured in biological fluids which has been regarded as the most reliable approach for accessing lipid peroxidation in vivo [48]. An alternate immunoassay (ELISA) for 8-isoprostaglandin F 2*α* in biological tissues is provided [49]. In recent years, the use of fluorescent probes specifically designed to detect oxidative stress in living cells, probes based on dihydrofluorescein diacetate, has been applied in vitro. A lipophilic fluorescent dye 4,4-difluoro-5-(4-phenyl-1,3-butadienyl)-4-bora-3a,4a-diaza-s-indacene-3-undecanoic acid (C11-BODIPY 581/591) to probe oxyl-radical-induced lipid oxidation by flow cytometry (FCM) has higher sensitivity and specificity for lipid peroxidation in vitro [50].

## 4. Roles of Lipid Peroxidation in Different Cell Death

### 4.1. Lipid Peroxidation in Apoptosis

Apoptosis is programmed series of events dependent on energy, as well as morphological features such as cell shrinkage, chromatin condensation, and presence of apoptotic bodies without inflammatory reactions [51, 52]. There are mainly three alternative pathways that lead to apoptosis: (1) extrinsic pathway, (2) intrinsic pathway, and (3) perforin/granzyme pathway. Caspases are key molecules involved in the transduction of the apoptosis signal, and all of the pathways converge to the executioner caspase-3 [53]. The extrinsic pathway is initiated by the tumor necrosis factor (TNF) receptor family interacting with a ligand and then binds with procaspase-8 following ligand-receptor interaction to activation of caspase-3 which leads to execution of apoptosis [54, 55]. The intrinsic pathway (mitochondrial pathway) employs alterations of inner mitochondrial membrane for induction of apoptosis. Apoptosis is triggered when the Bcl2-family proapoptotic proteins cause the opening of mitochondrial permeability transition pore and proapoptotic proteins into cytoplasm by interacting with apoptotic protease-activating factor 1 (Apaf-1) and procaspase-9 to constitute apoptosome [56, 57]. An assembly of apoptosome leads to caspase-9 activation, which further activates caspase-3, for apoptotic execution [58]. Perforin/granzyme-induced apoptosis is employed specifically by CD8+ cytotoxic T cells used by cytotoxic lymphocytes to eliminate virus-infected or transformed cells [59]. Granzyme B in the vesicles could activate procaspase-10 or directly activates caspase-3 for execution of apoptosis [60]. Granzyme A cleaves an inhibitory complex of a DNAse to induce apoptosis [61].

Lipid peroxidation play an important role in apoptosis. The products of lipid peroxidation interacts with membrane receptors and transcription factors/repressors to induce signaling for apoptosis. It can stimulate the activation of both the intrinsic and extrinsic apoptotic signaling pathways [62, 63]. ROS may lead to cardiolipin peroxidation, a mitochondrion-specific inner membrane phospholipid, and subsequent products of lipid peroxidation formation activated intrinsic apoptosis [64]. We next summarized different signal pathways of activation of apoptosis by the products of lipid peroxidation.

#### 4.1.1. The Products of Lipid Peroxidation Induce Apoptosis via Different Signal Pathways

The NF-*κ*B protein family is widely involve in inflammation, stress response, survival, and cell death [65]. Previous studies demonstrated that the product of lipid peroxidation increased NF-*κ*B activity by inhibiting I*κ*B degradation [66]. Besides the action of the product of lipid peroxidation on IKK, one of the NF-*κ*B pathway element was shown to phosphorylate antiapoptotic Bcl-2 for its inactivation upon lipid peroxidation [67]. It is also known that NF-*κ*B are also responsible for the transcriptional regulation of antiapoptotic expression [68]. Based on this background, lipid peroxidation was proposed to regulate the NF-*κ*B pathway, the antiapoptotic Bcl-2, and the crosstalk between these survival elements.

Mitogen-activated protein kinases (MAPKs) are also responsible for cellular signal transduction in response to a diverse set of stimulators including oxidative stress [69]. The activation of MAPKs causes the phosphorylation of serine, threonine, and tyrosine residues of proteins to executive regulating function. The extracellular signal-regulated kinase (ERK), p38, and Jun N-terminal kinase (JNK) can activate MAPKs under various conditions which affects cytoprotective or apoptotic signaling [70]. It has been shown that the product of lipid peroxidation forms adducts with ERK, JNK, and p38 to activate MAPKs for the activation of caspase signal initiating the apoptotic processes [71–73].

The protein kinase C (PKC) is a key regulator of a plethora of the transduction of cellular signals that regulate cell proliferation, differentiation, and apoptosis [74]. PKC isoforms are activated by growth factors by the stimulating phospholipase C (PLC), which generates inositol trisphosphate (IP3) and diacylglycerol (DAG) [75]. Many PKC isoforms are lipid-sensitive and Ca^2+^-dependent enzymes. The product of lipid peroxidation stimulates PKC indirectly through the activation of phospholipase C or affecting the activity of its subunits [76]. It has been suggested that the product of lipid peroxidation can activate protein kinase C-delta (PKC*δ*), a member of the lipid-regulated serine/threonine PKC family, preventing triglyceride accumulation in obese mice [77]. PKC*δ* is cleaved by caspase-3 to generate a constitutively activated catalytic fragment, which amplifies apoptosis cascades [78]. Thus, lipid peroxidation can activate the PKC pathway to regulate apoptosis.

### 4.2. Lipid Peroxidation in Autophagy

Autophagy, the process of cellular self-eating, is an important protein degradation pathway, especially during stress conditions. It is known as a cellular catabolic pathway that plays crucial roles in cellular homeostasis including the maintenance of cellular function and viability [79]. There are three main autophagic pathways: macroautophagy, microautophagy, and chaperone-mediated autophagy (CMA) [80, 81]. Chaperone-mediated autophagy involves the direct translocation of cytosolic proteins across the lysosomal membrane while microautophagy involves inward invagination of lysosomal membrane delivering a small portion of cytoplasm into the lysosomal lumen [82]. Among the three types of autophagy, the most extensively studied is macroautophagy which is mediated by a special organelle termed the autophagosome. During the process, light chain 3 (LC3) is involve in the formation of autophagosomes in mammalian cells that serves as a biomarker for occurrence of autophagy [83]. A selective form of macroautophagy in which mitochondria are specifically targeted for degradation at the autophagolysosome is so called mitophagy [84]. Mitophagy plays an essential role in cell differentiation, programming, cell death, and immune response [85]. Mitochondrial damage and dysregulation of mitophagy have been implicated in neurodegenerative diseases, cancer, and cardiac disease [86–88]. Autophagy helps to remove the damaged mitochondria and oxidized proteins, in most cases, supports survival. Under normal conditions, ROS-induced autophagy reduces damage caused by oxidative stress to protect cells. For instance, autophagy plays a protective role by eliminating ROS so as to preserve the integrity of mitochondria, prevent apoptosis, and promote antigen presentation. However, excessive autophagy induced by ROS can also cause autophagic cell death under certain circumstances [89].

A complex and differential regulation of autophagy by lipid peroxidation has been suggested by several studies [90–92]. The products of lipid peroxidation can adduct to specific mitochondrial and autophagy-related proteins driving cellular dysfunction in an autophagic cell death way [90]. During myocardial ischemia and reperfusion, autophagy signaling such as AMP-activated protein kinase and Akt-mTOR signaling is compromised by the products of lipid peroxidation through interference with upstream regulators [91]. Lipid peroxidation products may induce lysosomal dysfunction and lipofuscinogenesis which results in reduced autophagy activity [92]. We will summarize different signal pathways of activation of autophagy by the products of lipid peroxidation.

#### 4.2.1. The Products of Lipid Peroxidation Trigger Autophagic Cell Death via Different Signal Pathways (Figure 1)

The AMPK/mTORC pathway initiates autophagy. Adenosine monophosphate-activated protein kinase (AMPK) as upstream regulators of the mammalian target of rapamycin (mTOR) pathway senses nutrient and energy depletion and activates the tuberous sclerosis complex (TSC1–TSC2), leading to mTOR inactivation and initiation of autophagy [93]. The mammalian TOR (mTOR) pathway which negatively regulates macroautophagy is present in all types of lysosomes [94]. Inhibiting mTORC1 signaling by rapamycin, a major sensitive inhibitor of mTORC, significantly increased the level of LC3-II and led to autophagy [95]. It is suggested that the production of lipid peroxidation may activate mTORC1 signaling through direct inhibition of AMPK. The production of lipid peroxidation could conjugate with liver kinase B1 (LKB1), an upstream substrate of AMPK, and thus result in the activation of the mTOR pathway in isolated cardiomyocytes [96].

The JNK-Bcl-2/Beclin 1 pathway initiates autophagy. C-Jun N-terminal protein kinase (JNK), a member of the MAPK family, mediates Bcl-2 phosphorylation and Bcl-2 dissociation from Bcl-2/Beclin 1 complex which functions in the lysosomal degradation pathway of autophagy [97, 98]. JNK activation-induced phosphorylation of Bcl-2 plays an important role in Bcl-2-dependent autophagy without inactivation of the mTOR pathway which constitutes a distinct molecular signature of autophagy [99]. The production of lipid peroxidation could promote its interaction with JNK as a result of the nuclear translocation of this kinase to stimulate autophagy [100].

### 4.3. Lipid Peroxidation in Ferroptosis

Ferroptosis is a new form of programmed cell death characterized by iron-dependent increase in ROS [101]. Ferroptosis plays crucial roles in cellular proliferation, senescence, and differentiation. A study has shown that senescent cells were highly resistant to ferroptosis due to iron accumulation [102]. A specific ferroptosis inhibitor, ferrostatin-1 (Fer-1), has been used to evaluate the role of ferroptosis in various pathophysiological settings. Fer-1 could prevent oxidative lipid damage and could delay cyst development in polycystic kidney disease, suggesting the necessity of ferroptosis for cell proliferation in fibrosis-related disease [103]. Deletion of the *Gpx4*, one of the ferroptosis-executing gene, neuron-like cell became more sensitive to ferroptosis upon differentiation. These results reinforce the susceptibility of neuronal context to ferroptosis and suggest the value of ferroptosis in neuroprotection [103].

Ferroptosis can be triggered by structurally diverse small molecules (e.g., erastin, sulfasalazine, and RSL3) and also prevented by lipophilic antioxidants (CoQ10, Vitamin E, ferrostatins, and liproxstatins) [4, 8, 39, 104]. Ferroptosis occurs as a result of increased ROS levels due to elevated intracellular iron concentration and a depletion of antioxidant GSH that cause lipid peroxidation and consequently to cell death [101]. More and more studies have confirmed that GPX4 activity decreases or iron excess leads to ferroptosis [105–107]. Ferroptosis is distinct from apoptosis, autophagy, and other modes of cell death. How lipid peroxidation leads to ferroptosis is still an unsolved mystery [108]. Thus, in this review, we only focus on the mechanism of lipid peroxidation during ferroptosis related to both GPX4 activity and iron metabolism.

#### 4.3.1. GPX4 Activity Affecting Lipid Peroxidation Leads to Ferroptosis

GPX4 is an antioxidant enzyme that neutralizes lipid peroxides and protects membrane fluidity by using glutathione, as a cofactor of GPX4, to protect cells and membranes against peroxidation. Oxidized glutathione disulfide (GSSG) is subsequently reduced by glutathione reductase and NADPH/H+ to recirculate reduced glutathione (GSH) [109]. Inhibiting GPX4 can lead to increased ROS [110], while overexpression of GPX4 can reduce ROS and subsequently prevent cell from ferroptosis [111, 112]. GPX4 is a specific and robust central regulator of ferroptotic cell death when it is directly inhibited or indirectly inactivated by depletion of glutathione [112] (Figure 1).

#### 4.3.2. GPX4 Inhibitors and Selenium Influence GPX4 Activity Directly

RSL3 is a GPX4-specific inhibitor. Analysis of mass spectrometry-based proteomic data from an affinity pull-down experiment ranked GPX4 (PHGPx) as the top protein target for RSL3 [112, 113]. Inhibiting GPX4 by RSL3 generates lipid ROS and induces ferroptosis [114]. Selenium-containing GPX4 is important for living cell to allow the utilization of biomembranes for increased cellular plasticity and for the utilization of peroxides as second messengers in redox signaling processes [115, 116]. Without selenium, GPX4 lost its activity and cells are highly sensitive to oxidative damage due to irreversible overoxidation of the catalytically active-site thiolate [117]. FIN56 could decrease GPX4 abundance and derived production of coenzyme Q10. Studies have shown that FIN56 is not a cystine/glutamate antiporter inhibitor because it does not affect GSH levels. Instead, FIN56 treatment resulted in loss of GPX4 protein through posttranslational degradation and blocked mevalonate-derived production of lipophilic antioxidants such as coenzyme Q10 [118] (Figure 1).

#### 4.3.3. GSH Influence GPX4 Activity Indirectly

GPX4 plays an antioxidant effect by catalyzing its substrate—GSH. Thus, GSH influence GPX4 activity indirectly. Glutathione biosynthesis catalyzed by GCL (glutamate-cysteine ligase) and GS (glutathione synthetase) is essential for maintaining redox homoeostasis which needs cysteine, glutamate, and glycine as substrates [119]. Thus, the intracellular concentration of these substrates and the activity of enzymes affect GSH production.

Inhibiting cystine-glutamate antiporter decreases the intracellular concentration of cysteine which affects levels of GSH. The cystine/glutamate antiporter solute carrier family 7 member 11 (SLC7A11; also known as xCT) is a component of a plasma membrane transporter which is responsible for extracellular cystine and intracellular glutamate exchanging [120]. Erastin abolished the import of cysteine which is a precursor for glutathione during ferroptosis, and it was also proved to be a potent, selective inhibitor of system xc^−^ [4, 121]. Sorafenib, a multikinase inhibitor, is an FDA-approved drug used for treating advanced hepatocellular carcinoma [122]. Compared to other kinase inhibitors, sorafenib is the only drug that displays ferroptotic efficacy [123]. Similar to erastin, sulfasalazine had also been repurposed to induce ferroptotic cancer cell death via increased accumulation of lipid ROS [124].

Glutamate decreases the intracellular concentration of cysteine mediating ferroptosis. Glutamate induces oxidative stress via the inhibition of cysteine transporter xCT, leading to depletion of the cellular glutathione pool [125]. Inhibiting glutamate-induced toxicity can be initiated by calcium influx after glutamate receptor activation [126] or by competitive inhibition of a systemxc-dependent process, suggesting that ferroptosis is involved [127] (Figure 1).

Buthionine sulfoximine (BSO) induced ferroptosis by suppressing glutathione levels [113]. BSO is an inhibitor of glutamate-cysteine ligase, the rate limit in genzyme for glutathione synthesis [128]. Glutathione depletion causes loss of cellular antioxidant capacity and inhibition of glutathione-dependent enzymes such as glutamate-cysteine ligase. BSO was demonstrated to suppress glutathione levels and induce ferroptosis by inhibiting GCL [128] (Figure 1).

#### 4.3.4. The Production of ROS during Iron Metabolism Causes Lipid Peroxidation

Iron is required for numerous critical processes such as DNA synthesis, heme synthesis, and iron-sulfur cluster synthesis [129, 130]. It also plays an important role in the active sites of various enzymes which are involved in the formation such as LOX, xanthine oxidase, NADPH oxidases, and mitochondrial complex I and III [131–133]. However, the levels of iron in the cell need to be tightly balanced, as an excess of iron can impair cellular functions due to the generation of ROS and eventually cell death [106]. The increases of ROS caused lipid peroxidation and ferroptosis, which was suppressed by the treatment with iron chelator deferoxamine [8]. In addition, a higher level of iron transport proteins increased iron-mediated ROS and subsequently led to ferroptosis [134, 135]. Iron-mediated ROS was important to ferroptosis, and the production of ROS during iron metabolism was mainly from the process of “Fenton reaction” and *Haber-Weiss* reaction [8, 9]. Additionally, cells contain small amounts of uncoordinated and redox-active Fe^2+^, the so-called “labile iron pool” (LIP) [136]. Lysosomes can recycle endogenous iron sources like ferritin and mitochondria, and it is also a particularly large LIP [137]. Thus, lysosomes play a great important role in iron metabolism. Inhibitors of iron metabolism and iron chelators (e.g., deferoxamine (DFO) and ciclopirox (CPX)) suppress lipid peroxidation by reducing the availability of iron from iron pool [8]. Accordingly, both Fenton chemistry and iron-dependent enzymes may generate the reactive forms of oxygen that can trigger lipid peroxidation and finally cause ferroptotic cell death (Figure 1).

## 5. Lipid Peroxidation in Critical Illness

Critical illness including acute kidney injury, severe sepsis, and cardiac injury is interwoven with inflammation and oxidative stress, and the consequent production of lipid peroxidation plays an important role in the progression of disease. Abundant experimental and clinical data supported the important role of lipid peroxidation-related mechanisms in critical illness by reducing or genetic mutation to modulate lipid peroxidation. Inhibiting lipid peroxidation by Fer-1 can prevent folic acid- (FA-) induced acute kidney injury in mice which associates with downregulation of glutathione metabolism proteins, features that are typical of ferroptotic cell death [138]. Inactivation of the GPX4, a ferroptosis regulator to alleviate lipid peroxidation, triggers acute renal failure in mice suggesting that genetic mutation to modulate lipid peroxidation plays an important role in the pathological condition [139]. A study reported that propofol could protect against sepsis-induced liver dysfunction through suppressing hepatic oxidative stress and lipid peroxidation [140]. Carnosine, an endogenous histidyl dipeptides, protects cardiac myocytes against lipid peroxidation products of HNE and acrolein toxicity by directly reacting with these aldehydes [141].

As lipid peroxidation promotes the development and progression of critical illness, scientist tried to find strategies to treat/prevent it. However, in the current clinical practice, there is a lack of standardized preventive measures. As regards interventional measures against lipid peroxidation damage in critically ill patients, the interest has been focused on phospholipase A_2_ (PLA_2_), cyclooxygenase, and lipoxygenases which is an enzyme involved in the formation of lipid peroxidation [142]. The supplementation of antioxidants in critically ill patients such as vitamin C and E and selenium separately has been found to improve survival and prevent progressive organ dysfunction [143, 144]. As for complex pathological reasons or limited test method, clinical trials of these agents against lipid peroxidation in critical illness have partially failed [145, 146]. As regards for future perspective, detecting techniques targeted to lipid peroxidation biomarkers have been developed. The optimization of understanding the mechanisms of lipid peroxidation has gained a lot of interest, as well as the enhancement of their clinical intervention.

## 6. Summary and Perspectives

**Figure 4:**
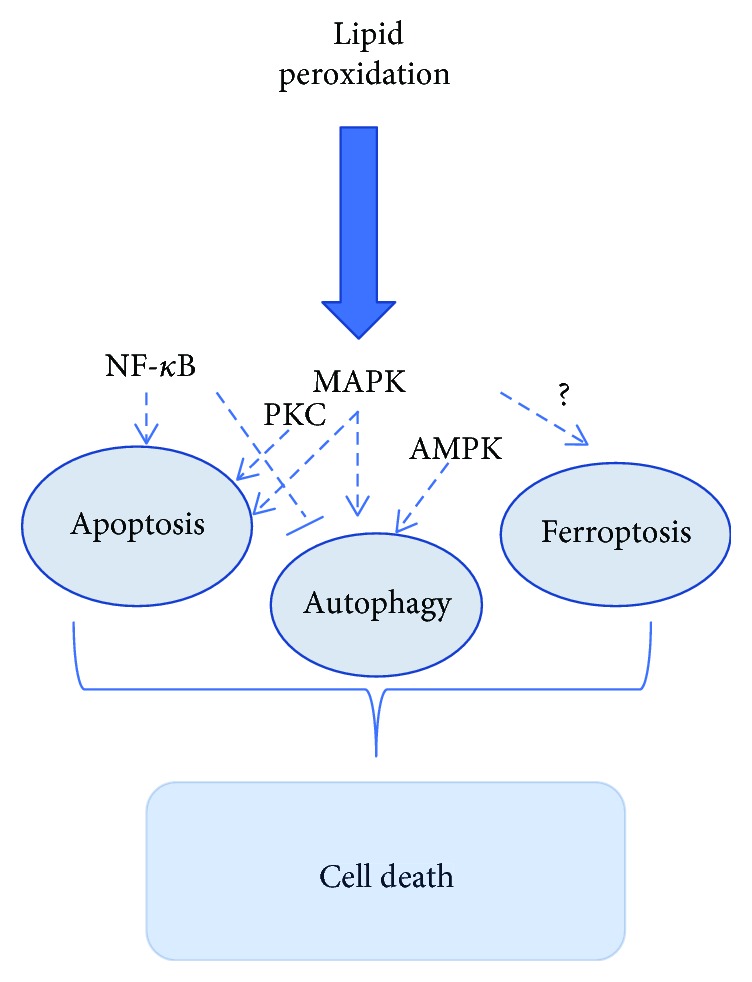
The relationship among ROS, lipid peroxidation, and cell death.

The connectivity of ROS-induced lipid peroxidation caused by apoptosis, autophagy, and ferroptosis manifests themselves in a seamless balance between life and death in response to cellular stress (Figure 4). The generation and elimination of ROS maintain the delicate balance, and the imbalance is associated with various pathologies such as cell proliferation, differentiation, and death. The general endogenous antioxidant system consisting of enzymatic antioxidants (SOD, CAT, GPx, and Trx) and nonenzymatic antioxidants (vitamins or its analogs, minerals, and metabolites) protects cell from oxidative damage. An excess of ROS induces lipid peroxidation via nonenzymatic (iron-dependent) and enzymatic (LOX-catalyzed) pathways which leads to cell death. According to species of lipid peroxidation products, UV/visible spectrophotometry, GC/MS, and ELISA can be used in vivo and C11-BODIPY 581/591 flow cytometry can be applied in vitro for detecting. The products of lipid peroxidation initiate apoptosis in different pathways (NF-*κ*B, MAPK, and PKC) and autophagy by AMPK/mTORC and JNK-Bcl-2/Beclin 1. Ferroptosis is a new form of programmed cell death noticed since 2012 [4]. In recent years, many studies had confirmed that ferroptosis was caused by loss of activity of the GPX4. GPX4 is normally functioned to remove the dangerous products of iron-dependent lipid peroxidation [4, 101, 104]. Although the products of lipid peroxidation have been extensively studied and excessive accumulation of lipid peroxidation has been shown to promote apoptosis and autophagy, their role in ferroptosis is unclear [66, 71, 108]. If the mysteries of lipid peroxidation-induced ferroptosis are solved, new insights and therapeutic strategies for ferroptosis-related human diseases can be provided.

## Abbreviations

ROS: Reactive oxygen species
PUFAs: Polyunsaturated fatty acids
NOXs: NADPH oxidases
XO: Xanthine oxidase
mETC: Mitochondrial electron-transport chain
SOD: Superoxide dismutase
CAT: Catalase
HNE: Hydroxynonenal
PL: Phospholipid
LOX: Lipoxygenase
Alox: Arachidonate lipoxygenase
PLA2: Phospholipase A2
GPx: Glutathione peroxidase
Trx: Thioredoxin
TrxR: Thioredoxin reductase
GSH: Glutathione
GSSG: Glutathione disulfide
CoQ10: Coenzyme Q10
HIF-1*α*: Hypoxia inducible factor-1*α*
MAPKs: Mitogen-activated protein kinases
ERK: Extracellular signal-regulated kinase
JNK: Jun N-terminal kinase
PKC: Protein kinase C
CMA: Chaperone-mediated autophagy
LC3: Light chain 3
mTOR: Mammalian target of rapamycin
AMPK: Adenosine monophosphate-activated protein kinase
TSC: Tuberous sclerosis complex
LIP: Labile iron pool
DFO: Deferoxamine
CPX: Ciclopirox.
